# SOCIAL ISOLATION AND FALLS RISK AMONG COMMUNITY DWELLING OLDER ADULTS: THE MEDIATING ROLE OF DEPRESSION

**DOI:** 10.1093/geroni/igz038.1078

**Published:** 2019-11-08

**Authors:** Jeffrey Burr, Lien Quach

**Affiliations:** 1 University of Massachusetts Boston, Boston, Massachusetts, United States; 2 Veterans Administration, Boston, Massachusetts, United States

## Abstract

Relatively little is known about the relationship between social isolation and the risk of falls among older adults. Yet, a considerable amount of research demonstrates that lack of sufficient social relationships, broadly defined, represents a modifiable risk factor for many indicators of well-being in later life. This study examines the association between two types of social isolation and the risk of falls. The study also examines whether depression mediates the association between social isolation and risk of falls. Longitudinal data from the Health and Retirement Study (2006-2012) were collected from community-dwelling participants aged 65 and older (N=8,464). The outcome variable was number of falls self-reported over the observation period. Independent variables included perceived isolation (feeling lonely, perceptions of social support), social disconnectedness (e.g., having no friends or relatives living nearby, living alone), and number of depressive symptoms. Results from regression models indicated that social disconnectedness was associated with a 5% increase in the risk of falls (IRR=1.05, 95% CI=1.01-1.09). Perceived social support was associated with a 21% increase in the risk of falls; when examined together, perceived social support and loneliness were associated with a combined 37% increase in falls risk. Depression was associated with a 47% increase in falls. Depression mediated the association between perceived isolation and falls. Further, perceived isolation mediated the association between social disconnectedness and falls. Reducing perceived social isolation and social disconnectedness may be an avenue for designing interventions to reduce the risk of falls, especially for older adults with depression.

